# Regulatory Roles of Histone Deacetylation in Metabolic Stress-Induced Expression of Caspase Recruitment Domain-Containing Protein 9 (CARD9) in Pancreatic β-Cells

**DOI:** 10.3390/ijms242115994

**Published:** 2023-11-06

**Authors:** Mirabela Hali, Nelson Pinto, Noah Gleason, Anjaneyulu Kowluru

**Affiliations:** 1 Biomedical Research Service, John D. Dingell VA Medical Center, Detroit, MI 48201, USA; mhali@med.wayne.edu (M.H.); hm7741@wayne.edu (N.P.); gp8205@wayne.edu (N.G.); 2 Department of Pharmaceutical Sciences, Eugene Applebaum College of Pharmacy and Health Sciences, Wayne State University, Detroit, MI 48201, USA

**Keywords:** histone deacetylase inhibitors, CARD9, pancreatic islet β-cell, TSA and SAHA

## Abstract

CARD9, a scaffolding protein, has been implicated in the pathogenesis of metabolic diseases, including obesity and diabetes. We recently reported novel roles for CARD9 in islet β-cell dysregulation under duress of gluco (HG)- and glucolipotoxic (GLT) stress. CARD9 expression was also increased in β-cells following exposure to HG and GLT stress. The current study is aimed at understanding the putative roles of histone deacetylation in HG- and GLT-induced expression of CARD9. Using two structurally distinct inhibitors of histone deacetylases (HDACs), namely trichostatin (TSA) and suberoylanilide hydroxamic acid (SAHA), we provide the first evidence to suggest that the increased expression of CARD9 seen under duress of HG and GLT stress is under the regulatory control of histone deacetylation. Interestingly, the expression of protein kinase Cδ (PKCδ), a known upstream regulator of CARD9 activation, is also increased under conditions of metabolic stress. However, it is resistant to TSA and SAHA, suggesting that it is not regulated via histone deacetylation. Based on these data, we propose that targeting the appropriate HDACs, which mediate the expression (and function) of CARD9, might be the next step to further enhance our current understanding of the roles of CARD9 in islet dysfunction under metabolic stress and diabetes.

## 1. Introduction

Many biological processes, including the expression of inflammatory genes, DNA repair, and cell proliferation, are governed by modifications at the genomic level. The two key mechanisms regulating gene transcription are DNA methylation and histone acetylation. Histone acetyltransferases (HATs) and histone deacetylases (HDACs) represent two classes of enzymes, which control gene transcription by regulating the overall acetylation status of histones, thereby rendering modification of the chromatin structure for either activation or repression of transcription. HATs acetylate the ɛ-amino acid groups of lysine residues of histones. The addition of acetyl groups neutralizes the positive charges and increases the hydrophobicity of histones, thereby reducing their affinity to DNA, and altering the nucleosome structure, which facilitates the binding of transcriptional factors. The HDACs reverse the function of HATs by removing the acetyl groups from the acetylated histones. A growing body of recent evidence suggests that transcriptional dysregulation due to imbalances between the functional activation of HATs and HDACs affects cellular functions, including cancer, fibrosis-associated diseases, diabetes, and associated complications [1,2,3,4,5,6,7,8,9].

Caspase recruitment domain-containing protein 9 (CARD9) is a scaffolding protein, which is expressed abundantly in macrophages, dendritic cells, monocytes, and neutrophils [10,11,12,13,14,15,16,17,18]. Recent evidence in animal models with CARD9 deletion demonstrated roles for CARD9 in diet-induced inflammation, obesity, and metabolic pathologies [19,20,21]. Cao and coworkers demonstrated that CARD9 knockout ameliorates myocardial dysfunction associated with high-fat-diet-induced obesity via the suppression of p38 MAPK phosphorylation and preservation of autophagy in the heart [20]. Peterson and coworkers reviewed evidence in support of the postulation that CARD9 might be a potential therapeutic target for cardiovascular disease [22]. In this context, recent evidence from our laboratory indicated that CARD9 is expressed in human islets, rat islets, mouse islets, and INS-1 832/13 cells. We also demonstrated a significant increase in the abundance of CARD9 in β-cells following exposure to diabetogenic conditions (e.g., gluco- and glucolipotoxicity) [23]. Lastly, findings from these studies also revealed that knockdown (siRNA) of CARD9 markedly inhibited HG-induced activation of Rac1 and p38MAPK in pancreatic β-cells. Together, these findings implicated novel roles for CARD9 in islet dysregulation under hyperglycemic stress, further affirming roles for CARD9 in the pathology of metabolic diseases [19,20,21].

Published evidence implicates protein kinase Cδ (PKCδ) in the onset of islet β-cell dysregulation following exposure to a variety of metabolic stress conditions [24,25,26,27]. For example, studies by Hennige and coworkers demonstrated that overexpression of kinase-negative PKCδ in pancreatic β-cells protects mice from diet-induced glucose intolerance and β-cell dysfunction [26]. In the context of the current investigation, it has been shown that PKCδ mediates the phosphorylation of CARD9, leading to its functional activation [28,29,30,31]. Based on the evidence that the PKCδ-CARD9 signaling module might contribute to islet β-cell dysregulation, we investigate herein the putative roles of the histone deacetylation signaling step on the expression of CARD9 and PKCδ in pancreatic β-cells exposed to diabetogenic conditions. We address this question via a pharmacological approach involving two structurally distinct inhibitors, namely TSA and SAHA, on the metabolic stress-induced expression of CARD9 and PKCδ in insulin-secreting INS-1 832/13 pancreatic β-cells. Data from these investigations revealed differential regulatory roles of histone (de)acetylation steps in the increased expression of CARD9 and PKCδ in pancreatic β-cells following exposure to metabolic stress.

## 2. Results

**Figure 1:**
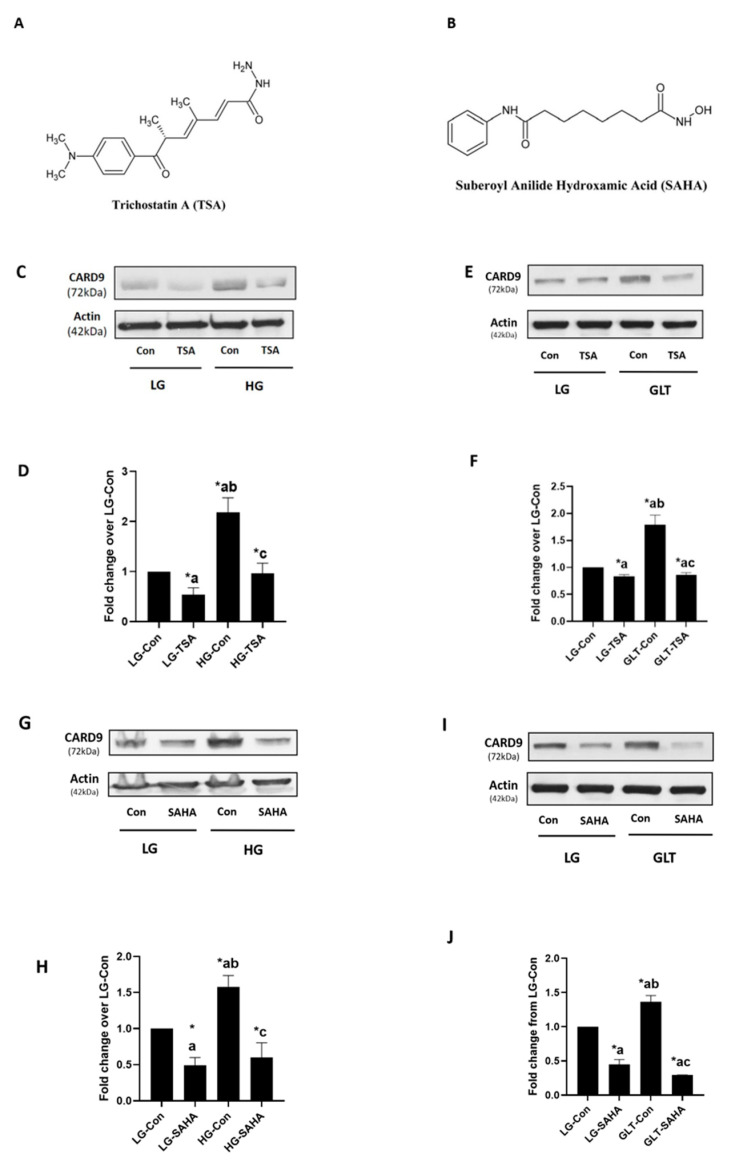
HG- or GLT-induced expression of CARD9 is inhibited by TSA and SAHA in INS-1 832/13 cells: Chemical structures of TSA (**A**) and SAHA (**B**) used in the current investigations are shown here. Cells were cultured under LG or HG conditions with TSA (250 nM) or DMSO (diluent control) for 24 h. Abundance of CARD9 and β-Actin (loading control) was determined with Western blotting. A representative blot from four studies is shown in (**C**), and densitometric quantification of the pooled data is provided in (**D**). Results are expressed as mean ± SEM. * *p* < 0.05. a: vs. LG-Con; b: against LG-TSA; and c: vs. HG-Con. Cells were treated with LG (2.5 mM) or GLT conditions (20 mM glucose plus 500 µM palmitate) with TSA (250 nM) or DMSO (diluent control) for 24 h. CARD9 expression was determined with Western blotting (**E**). A representative blot from three independent studies is shown here. (**F**) Densitometry analysis was carried out on CARD9 bands and the ratios of CARD9: β-Actin are provided herein. Pooled data from the three experiments were plotted as fold change from the LG-Control treated group (LG-Con). The results are indicated as mean ± SEM. * *p* < 0.05. a: vs. LG-Con; b: vs. LG-TSA; c: compared with GLT-Con. (**G**) Relative abundance of CARD9 in lysates from cells exposed to either LG or HG conditions with SAHA (100 µM) or DMSO (diluent control) for 24 h was determined with Western blotting. A representative blot from three studies is shown here. (**H**) Combined data accrued from the three studies were plotted. * *p* < 0.05. a: compared with LG-Con; b: compared with LG-SAHA; c: vs. HG-Con; (**I**) INS-1 832/13 cells were treated with LG or GLT conditions without or with SAHA (100 μM) or DMSO (diluent) for 24 h. Abundance of CARD9 was assessed with Western blotting. A representative blot from two independent studies is shown here. (**J**) Pooled data from the two experiments were plotted. The results are expressed as mean ± SD. * *p* < 0.05. a: compared with LG-Con; b: compared with LG-SAHA; c: compared with GLT-Con.

Recent studies from our laboratory reported higher levels of CARD9 in rodent and clonal β-(INS-1 832/13) cells exposed to glucotoxic (20 mM; 24 h HG), lipotoxic (500 µM palmitate; 24 h LT) and glucolipotoxic (a combination of glucose and palmitate; GLT) environments. Mannitol (20 mM; 24 h), used as an osmotic control, exerted no effects on the expression of CARD9 in these cells; these findings suggest that the effects of HG and GLT conditions might underlie their metabolic regulation of CARD9 expression, not due to osmolality [23]. As stated above, in the current study, we examined the putative regulatory roles of histone deacetylation in CARD9 expression induced by HG and GLT conditions (referred to as metabolic stress throughout) in INS-1 832/13 cells. To address this question, we utilized two structurally distinct inhibitors of HDACs, namely TSA (Figure 1A) and SAHA (Figure 1B), on CARD9 expression induced under the conditions of metabolic stress.

### 2.1. TSA and SAHA Inhibit Metabolic Stress-Induced CARD9 Expression in INS-1 832/13 Cells

As we reported recently [23], the Western blot data depicted in Figure 1C demonstrate a significant increase in the expression of CARD9 in INS-1 832/13 cells under duress of HG exposure conditions. The co-provision of TSA (250 nM) significantly attenuated HG-induced expression of CARD9 in these cells. Aggregate data from four studies are depicted in Figure 1D. Next, we examined the effects of TSA on GLT-induced effects on CARD9 expression. The Western blot data provided in Figure 1E show a significant reduction in GLT-induced expression of CARD9 following co-provision of TSA. Collective data from these studies are shown in Figure 1F. Altogether, these findings suggest that hypoacetylation of histones is a requisite for HG- and GLT-induced expression of CARD9 in pancreatic β-cells.

In the next set of studies, we further validated the contributory roles of histone hypoacetylation on HG- and GLT-induced CARD9 expression using SAHA, a known inhibitor of HDACs, which is structurally distinct from TSA (Figure 1A,B). The findings highlighted in Figure 1G demonstrate a marked reduction in the abundance of CARD9 in cells exposed to SAHA under both basal and HG stress conditions. Pooled data from multiple studies are shown in Figure 1H. In a manner akin to its effects on basal and HG-induced CARD9 expression, the data highlighted in Figure 1I,J demonstrate a marked suppression, by SAHA, of basal and GLT-induced expression of CARD9 in these cells. Collectively, the findings highlighted in Figure 1C–J implicate novel roles for histone hypoacetylation in the sequence of events leading to the metabolic stress-induced expression of CARD9 in pancreatic β-cells (see Discussion below).

### 2.2. Hyperglycemic Conditions Significantly Increase the Expression of PKCδ in an HDAC-Independent Manner

**Figure 2:**
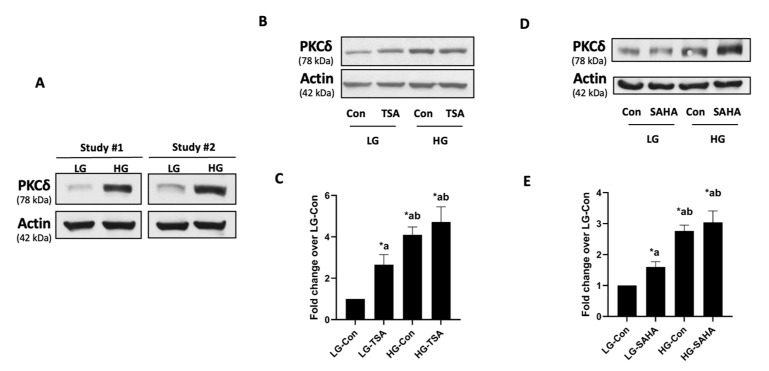
Increased expression of PKCδ seen under chronic hyperglycemic conditions is resistant to TSA or SAHA in INS-1 832/13 cells: (**A**) Cells were incubated (24 h) under LG or HG conditions. Expression of PKCδ in cell lysates was determined with Western blotting. Data from two studies are shown here. (**B**) Cells were cultured under LG or HG conditions in the presence of TSA (250 nM) or diluent (DMSO; 24 h). A representative Western blot (from 5 experiments) depicting the expression of PKCδ is provided here. (**C**) Densitometry analysis was conducted on PKCδ bands and the ratios of PKCδ: β-Actin are shown herein. Pooled values from the five studies are provided here. Data are expressed as mean ± SEM. * *p* < 0.05. a: compared with LG-Con; b: vs. LG-TSA. (**D**) Cells were exposed to LG or HG conditions with SAHA (100 μM) or DMSO (diluent control) for 24 h. Relative abundance of PKCδ was determined with Western blotting. A representative blot from four independent studies is shown here. (**E**) Combined data from four studies are shown. The results are indicated as mean ± SEM. * *p* < 0.05. a: compared with LG-Con; b: compared with LG-SAHA.

**Figure 3:**
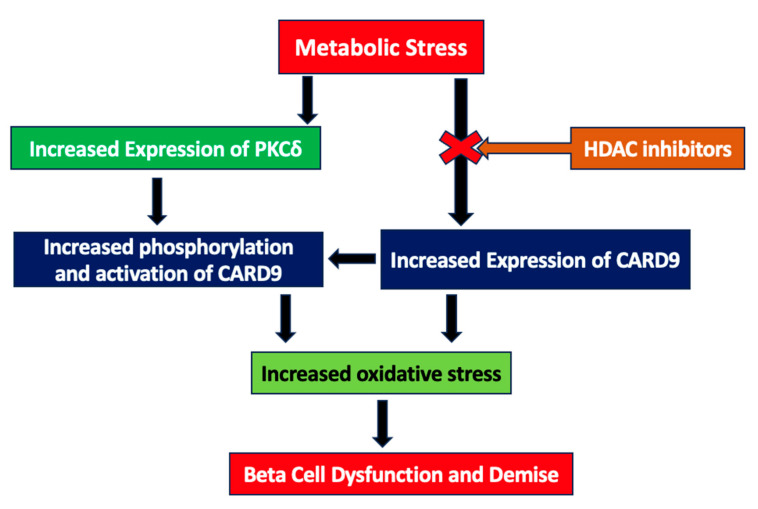
Schematic depiction of potential mechanisms underlying metabolic stress-induced expression of CARD9 and PKCδ in the islet β-cell: Potential regulatory roles of histone hypoacetylation. Based on these data, we propose that metabolic stress induces expression of CARD9 via increased deacetylation of CARD9; inhibition of these signaling steps by HDAC inhibitors (TSA and SAHA) culminates in the suppression of CARD9 expression under duress of metabolic stress. Putative effects of HDAC inhibition on metabolic stress-induced, CARD9-mediated generation of oxidative stress and p38 MAPK activation [23] remain to be examined further. In addition, based on our data, we propose that metabolic stress induces the expression of PKCδ in a histone deacetylation-independent manner, which, in turn, increases the phosphorylation and functional activation of CARD9. The latter needs further experimentation.

As stated above, published evidence suggests that the functional activation of CARD9 is mediated via phosphorylation. For example, it has been reported that both Dectin-1 and Dectin-2 elicit direct regulatory effects via Syk-phospholipase Cγ-mediated effects on PKCδ, which, in turn, phosphorylates CARD9 at Thr-234, leading to its functional activation [31]. Several lines of evidence in pancreatic β-cells suggest critical roles for PKCδ in the onset of islet β-cell dysfunction following exposure to diabetogenic stimuli [24,25,26,27]. Therefore, at the outset, we asked if hyperglycemic conditions exert any regulatory effects on PKCδ expression in a manner akin to CARD9 expression, and, if so, whether histone acetylation–deacetylation signaling steps could contribute to alterations in the expression of PKCδ in pancreatic β-cells under HG stress. To answer this question, we assessed the expression levels of PKCδ in β-cells under HG culture conditions (as in the studies highlighted in Figure 1). The data accrued in the studies shown in Figure 2A indicate a significant increase in the expression of PKCδ in these cells following exposure to HG conditions. Interestingly, in contrast to data accrued in CARD9 expression studies (Figure 1), co-provision of either TSA or SAHA significantly increased the expression of PKCδ under basal (LG) conditions without significantly exerting any effects on its expression induced under duress of hyperglycemia (Figure 2B–E). Together, these findings suggest differential regulation of histone hypoacetylation on the metabolic stress-induced expression of CARD9 and PKCδ in pancreatic β-cells (Figure 3 below).

## 3. Discussion

Evidence for the potential involvement of CARD9 in islet function in physiological insulin secretion, and under HG and GLT stress, is beginning to emerge. Recently, Gamage et al. [32] reported the expression of CARD9 in a variety of insulin-secreting cells (e.g., human islets, rodent islets, and clonal β-cells). The depletion of CARD9 (siRNA) significantly inhibited glucose-stimulated insulin secretion (GSIS), without significantly affecting glucose-induced Rac1 activation. Subsequent investigations have revealed that CARD9-mediated GSIS involves the activation of p38MAPK, not ERK1/2, phosphorylation in β-cells. Together, these studies have demonstrated that CARD9 is involved in GSIS via Rac1-independent and p38MAPK-sensitive signaling steps [32]. Follow-up investigations by these investigators have shown that CARD9 expression is significantly increased following exposure to metabolic stress conditions in mouse islets and clonal β-cells. Knockdown of CARD9 markedly suppressed HG-induced activation of Rac1 and phosphorylation of p38MAPK and *RelA*. Interestingly, however, no significant effects on the HG-induced phosphorylation of JNK1/2 and ERK1/2 were noted in β-cells in which CARD9 expression was depleted. Together, data accrued in these studies implicate novel roles for the Rac1-p38MAPK-NFκB signaling module in CARD9-mediated metabolic dysregulation of the β-cell under diabetogenic conditions [23].

One of the objectives of the current investigation was to determine if increased expression of CARD9 seen under metabolic stress conditions is regulated by histone (de)acetylation steps. Using a pharmacological approach, we demonstrate significant inhibition of HG- and GLT-induced CARD9 expression by TSA or SAHA, two known inhibitors of HDACs, in INS-1 832/13 cells. These findings provide the first evidence for the regulatory control of histone hypoacetylation in metabolic stress-induced expression of CARD9 in pancreatic β-cells. Whether HDAC-mediated inhibition of increased expression of CARD9 prevents the activation of signaling steps downstream to CARD9 activation (as above), leading to the restoration of normal islet β-cell function, including optimal GSIS, remains to be verified. Furthermore, additional investigations are needed for the identification of a specific HDAC that mediates the deacetylation of histones to impact CARD9 expression. It is noteworthy that our current findings lend support to earlier investigations demonstrating the utility of HDAC inhibitors in the prevention of islet β-cell dysfunction induced under various experimental conditions, both in vitro and in vivo. Using MS-275, a known inhibitor of class 1 HDAC, Plaisance and coworkers reported the inhibition of palmitate-induced cell demise in human islets and MIN6 β-cells [33]. Data from the studies of Remsberg et al. suggest that the genetic deletion of HDAC3 improves glucose tolerance in increased insulin secretion from adult β-cells [34]. Daneshpajooh and coworkers reported significantly high expression of HDAC7 in islets from T2DM donors. The pharmacological inhibition or siRNA-mediated knockdown of HDAC7 restored GSIS in pancreatic β-cells overexpressing HDAC7. Subsequent studies from these investigators demonstrated significant protection of mitochondrial dysfunction and apoptosis and restoration of glucose-stimulated insulin secretion in islets from T2DM donors [35]. Along these lines, Sonthalia and associates recently highlighted the importance of HDAC inhibitors as potential antidiabetic agents [36].

In the current study, we also observed increased expression of PKCδ in β-cells exposed to chronic HG stress. In this context, earlier investigations suggested that CARD9 activation is under the regulatory control of phosphorylation, specifically coupled to PKCδ activation [28,30,31,37]. It was demonstrated that Dectins (Dectin-1 and Dectin-2) elicit direct regulatory roles via Syk-phospholipase Cγ-mediated effects on PKCδ, which, in turn, phosphorylates CARD9 at Thr-234, culminating in its functional activation [31]. It has also been suggested that while phosphorylation of CARD9 at Thr-234 leads to its activation, phosphorylation at T531/T533 has been shown to attenuate CARD9 function [18]. The potential regulation of the functional activation of CARD9 via PKCδ-mediated phosphorylation in pancreatic β-cells under duress of metabolic stress remains to be investigated further. Interestingly, we observed no significant effects of TSA and SAHA on the HG-induced expression of PKCδ, thus suggesting that metabolic stress induces the expression of CARD9 and PKCδ via histone hypoacetylation-dependent (CARD9) and -independent (PKCδ) mechanisms (see below).

Based on these data, we propose a model (Figure 3) to implicate the positive modulatory role(s) of histone deacetylation (or hypoacetylation) in metabolic stress-induced CARD9 expression in INS-1 832/13 cells. The exposure of the pancreatic β-cell to HG and GLT stress results in the hypoacetylation of histones. The inhibitors of HDACs prevent such a signaling step by inhibiting the candidate HDAC, thereby retaining specific histones in a hyperacetylated state. This, in turn, halts the metabolic stress-induced expression of CARD9. The potential impact of HDAC inhibition on the metabolic stress-induced, CARD9-mediated generation of oxidative stress and stress kinase activation [23] remains to be investigated further.

The expression of PKCδ, a known upstream regulator of CARD9 activation, is also increased under conditions of metabolic stress. However, the increased expression of PKCδ seen under HG exposure conditions is resistant to HDAC inhibition, suggesting that it is not under the regulatory control of histone deacetylation. Interestingly, however, our current study demonstrates a significant increase in the expression of PKCδ in the presence of TSA and SAHA in pancreatic β-cells incubated with basal glucose. These data imply that histone hyperacetylation is a requisite for PKCδ expression in pancreatic β-cells. In this context, extant studies in neuronal cells demonstrated increased expression of PKCδ following exposure to a variety of HDAC inhibitors (e.g., sodium butyrate, TSA, valproic acid, and apicidin). Importantly, such effects appear to be specific to PKCδ, since the expression of other PKC isoforms (e.g., PKCα) was not impacted by HDAC inhibitors [38]. Likewise, studies by Wang and coworkers in human colorectal cancer cells have reported increased expression of PKCδ following the inhibition of histone deacetylation with MPT0G030, a novel class I HDAC inhibitor [39]. Therefore, it is likely that the increased expression of PKCδ that we observed in INS-1 832/13 cells, in the presence of TSA and SAHA, might underlie a similar mechanism. Additional studies are needed to validate this formulation in pancreatic β-cells.

From a translational standpoint, studies by Kaur and coworkers have reported contributory roles of CARD9 to human diabetes [40]. We envision steady progress in this field that would fill in the gaps in our current understanding of the CARD9-PKCδ signaling module in the onset of metabolic dysfunction in islet β-cell dysfunction and the loss of functional β-cell mass in diabetes. Based on our data, we also propose that metabolic stress induces the expression of PKCδ in an HADC-independent manner, which, in turn, increases the phosphorylation and functional activation of CARD9, although this remains to be verified experimentally. Lastly, data accrued in the current investigations raise a potential possibility for the use of HDAC inhibitors as novel therapeutics to prevent metabolic stress-mediated metabolic dysfunction in islet β-cells. Future studies will need to focus on the development of novel inhibitors with minimal cytotoxic effects, but with a significant inhibitory potency against candidate HDACs.

## 4. Materials and Methods

### 4.1. Materials

CARD antibody (catalog # sc-374569) was from Santa Cruz Biotechnology (Dallas, TX, USA). Antibodies directed against PKCδ (catalog # 2058), Mouse and Rabbit Secondary antibodies (HRP-conjugated) were purchased from Cell Signaling Technology (Danvers, MA, USA). Antibody for β-actin (catalog # A1978) was from Sigma Aldrich (St. Louis, MO, USA). TSA (catalog # 89730) and SAHA (catalog # 10009929) were obtained from Cayman Chemicals (Ann Arbor, MI, USA).

### 4.2. Culture of Insulin-Secreting INS-1 832/13 Cells

INS-1 832/13 cells were cultured in RPMI-1640 medium containing 10% FBS supplemented with 100 IU/mL penicillin and 100 IU/mL streptomycin, 1 mM sodium pyruvate, 50 µM 2-mercapto-ethanol, and 10 mM HEPES (pH 7.4). Prior to each study, cells were cultured (overnight) in low-serum (2.5% fetal bovine serum) and low-glucose (LG; 2.5 mM) medium. Cells were further treated in media containing low-glucose (LG; 2.5 mM), high-glucose (HG; 20 mM), or HG (20 mM glucose) and palmitic acid (PA; 500 µM) for 24 h in the absence or presence of HDAC inhibitors, namely TSA (250 nM) and SAHA (10 µM), as described in the text.

### 4.3. Western Blotting

INS-1 832/13 cell lysate proteins (30 µg protein) were resolved with SDS-PAGE and transferred onto nitrocellulose membranes. Following blocking (in 3% BSA; 1 h at room temperature), membranes were incubated (overnight) with the primary antibody (1:1000 dilution). The membranes were washed and probed with the appropriate secondary antibody (1:2000 dilution). The protein bands were identified (ECL kit; ThermoScientific, Waltham, MA, USA), and their intensities were calculated (Image Studio Lite imaging software version 3.1; LI-COR Biosciences, Lincoln, NE, USA).

### 4.4. Statistical Analysis

Data were analyzed using GraphPad Prism software version 9.5 (GraphPad Software, San Diego, CA, USA). Data are presented as mean ± standard error of mean (SEM) from three or more independent experiments. Comparisons between two groups were analyzed with a two-tailed Student *t*-test, while studies incorporating multiple groups were analyzed with a one-way analysis of variance (ANOVA) with Tukey’s multiple comparison. A *p* value below 0.05 was considered significant.

## Data Availability

Data will be made available on request.
